# Various Endoluminal Approaches Available for Treating Pathologies of the Aortic Arch

**DOI:** 10.1007/s00270-020-02561-y

**Published:** 2020-06-25

**Authors:** Muzaffar A. Anwar, Mohammad Hamady

**Affiliations:** 1 Department of Surgery and Cancer, Imperial College-London, London, England; 2 St Mary’s Hospital, Praed Street, London, W2 1NY England

**Keywords:** Aortic arch aneurysm, Total aortic arch replacement, Endovascular aortic arch repair, Chimney, Scallop, Branch stent graft

## Abstract

Open surgical repair of the aortic arch for degenerative aortic disease in an unfit patient is associated with significant morbidity and mortality. Endoluminal techniques have advanced over the last decade. Contemporary endovascular options including a hybrid approach (supra-aortic debranching and aortic stent graft), inner branched endograft, chimney stents, and scallop or fenestrated endografts are being used frequently as an alternative to open surgical arch repair. Understanding of the available endoluminal technology along with careful planning and effective teamwork is required to minimise complications associated with the endoluminal techniques, particularly neurological ones. Custom made techniques are superior to chimney or parallel technology in terms of their complications and durability. Integration of the protective devices such as embolic protection filters into stent design may reduce the risk of poor neurological sequelae. Long-term data are needed to assess the durability of these devices.

## Introduction

Over the last decade, significant progress has been made in the treatment modalities for the aortic arch diseases. Open surgery is still considered the gold standard in young and fit patients as well as in patients with genetic aortic syndrome. Open aortic arch repair requires cardiopulmonary bypass, deep hypothermal circulatory arrest and antegrade or retrograde cerebral perfusion. Cardiopulmonary comorbidities frequently exist in patients with degenerative aortic disease which increase the overall mortality and morbidity with the open repair. Comparable perioperative mortality and stroke rates have been reported between the endovascular arch repair and open arch repair, despite the older age and a higher comorbid profile of patients in the endovascular group [1]. Elective and emergency open repair of arch pathology carries mortality risk of 9% and 37%, respectively [2]. This review focuses on contemporary endovascular options for the treatment of aortic arch pathologies which include hybrid approach (supra-aortic debranching and stent graft), inner branched endograft, chimney stents, and scallop or fenestrated endografts.

**Table:** Fact Sheet: Key points to remember while planning endovascular aortic arch cases

1	Proximal landing zone should be at least 20 mm (measured on the inner curvature of aorta) and the aortic diameter < 38 mm
2	Arch angulation > 60°* and absence of thrombus in the sealing zone of the aortic arch
3	Use of stent graft in the native aorta of patients with connective tissue disease is not recommended
4	Access vessel should be > 7 mm in diameter
5	Stroke risk is high with endovascular approach especially in hostile anatomy—presence of thrombus/atherosclerotic plaque in the arch
6	Cardiac output would require to be reduced during the main stent deployment to avoid windsock effect and device migration. This can be achieved either via rapid overdrive cardiac pacing or via pharmacological means
7	Retrograde dissection is a recognised complication and is more common in cases where diameter is more than 38 mm
8	Spinal cord ischemia is a potential serious complication, especially in cases where left subclavian artery is sacrificed. Prophylactic CSF pressure monitoring and drainage especially in cases of extensive aortic coverage with a target CSF pressure between 10 and 12 mmHg is considered a protective measure
9	Unfractionated heparin is needed during endovascular repair to prevent risk of thrombotic complications. Dose of the heparin can be variable depending on factors such as patient’s weight, underlying thrombotic/ bleeding risks and duration of the procedure. Activated clotting time (ACT) should be performed in theatre. ACT target of > 250 is considered an accepted marker for patient’s anti thrombotic status
10	Assessment and patency of circle of willis prior to endoluminal intervention is advisable
**Important numbers in endoluminal techniques**	
Proximal landing zone length	20 mm
Native aortic diameter	< 38 mm
Arch angulation	> 60°
Access vessel for the main device	> 7 mm
Ascending aortic length (from sinotubular junction to origin of innominate)	> 45 mm
**Important studies**	
Haulon S et al. 2014 [15]	Global experience with an inner branched arch endograft. *J Thorac Cardiovasc Surg*. 2014; 148: 1709–16
Ahmad W et al. 2017 [24]	A current systematic evaluation and meta-analysis of chimney graft technology in aortic arch diseases. *J Vasc Surg*. 2017; 66: 1602–10 e2
Cao P et al. 2012 [10]	De Rango P, Czerny M, et al. Systematic review of clinical outcomes in hybrid procedures for aortic arch dissections and other arch diseases. *J Thorac Cardiovasc Surg*. 2012; 144: 1286–300, 300 e1-2
Tsilimparis et al. 2019 [16]	Single-center experience with an inner branched arch endograft. *J Vasc Surg*. 2019; 69: 977–85 e1
**Two messages**	
1	Careful planning, excellent familiarity with the device and an established coordinated team-work are keys to achieve better outcomes from endoluminal techniques in high risk patient with aortic arch disease
2	Custom made branched, fenestrated or scalloped endoluminal techniques should be preferred over the chimney technique for elective repair of aortic arch. Chimney technique should be reserved as a bailout approach or in emergency cases only
**Prediction for the Future**	
In the years to come, endoluminal techniques will increasingly be used in treating aortic arch pathology. Further refinement of stent graft material and characteristics is necessary, including cerebral protection devices integrated into the endograft device, proved measures to reduce air emboli and lower profile of delivery system and bridging stents	

*Angulation is measured by the type of the arch and the radius of curvature (ROC) which is measured at the closest edge of wall of ascending aorta to adjacent closest edge of descending thoracic aorta (at the level of the pulmonary artery bifurcation) [37]

## Indications for Aortic Arch Intervention

A range of aortic arch pathologies can be treated with endoluminal techniques including complicated aortic dissection, aortic arch aneurysms, penetrating aortic ulcer and intramural haematoma (progressive or symptomatic). Intervention is indicated for elective repair of isolated arch aneurysm at 5.5 cm in size [3] and annual growth rate of > 5 mm per year [4]. Classically, uncomplicated acute Stanford type B dissection is managed medically with blood pressure control. Intervening during the acute or subacute phase remains less well defined and beyond the scope of this review. Complicated aortic dissections characterised by rapid expansion of the aortic diameter, persistent chest pain despite optimum medical treatment, evidence of limb or visceral malperfusion, paraplegia or paraparesis, or hypertension refractory to medical management dictate urgent intervention. Intramural haematoma (IMH) is considered a precursor for aortic dissection and may necessitate intervention. Isolated Type A (ascending aorta) IMH is treated with open surgery, while intervention for type B (arch and descending aorta) is indicated if there is a progression of symptomatic aortic wall thickness over short interval scans. Ulcerated atherosclerotic plaque could lead to the development of penetrating aortic ulcer (PAU). PAU constitutes around 2–7% of all acute aortic syndrome and may require intervention. In our institution, intervention is considered for large PAU (> 3.75 cm) and/or symptomatic PAU associated with IMH, persistent or recurrent chest pain, contained rupture, and presence of pleural effusion. Initial ulcer diameter of > 20 mm or the ulcer depth of > 10 mm is considered high risk for propagation [3, 5].

## Key Principles to Remember While Planning Endoluminal Intervention for Aortic Arch

**Fig. 1:**
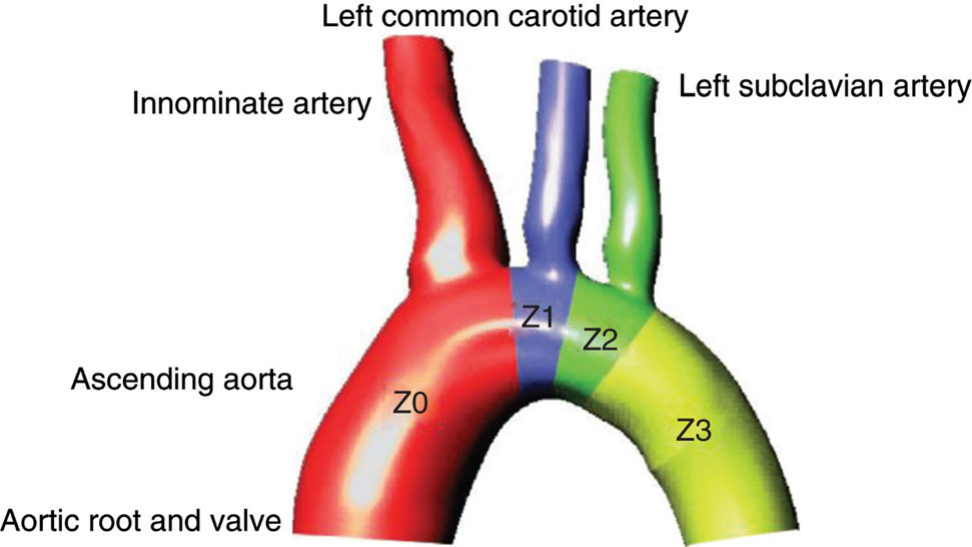
Published with permission from John Wiley and sons. “Ishimura classification of zones of the aortic arch: zone (Z) 0, ascending aorta to innominate artery; Z1, innominate artery to left common carotid artery; Z2, left common carotid artery to subclavian artery; Z3, left subclavian artery to proximal descending thoracic aorta”

The complexity of stent design, anatomical difficulties, the inherent high risk of neurological complications and complex hemodynamic forces in the ascending aorta and aortic arch are the main challenges facing endovascular intervention in this part of the body. Thorough anatomy assessment and careful planning of the procedure using high-quality cross-sectional computed tomography angiography with thin axial slices for multiplanar reconstruction are essential prerequisite for planning complex arch procedures. This should be accompanied by a well-coordinated teamwork preparation. Adequate access vessels for the main device and for fenestration or branched or chimney stent placement are crucial. Femoral access is used for the deployment of the main arch device. Heavy calcifications in the iliac or femoral arteries may restrict the delivery of the stent graft. Construction of an iliac conduit may be required to allow the safe passage of a 20–25 French sheath. Percutaneous or open femoral access is at the discretion of the operator, but a scarred groin from previous surgery may make access challenging. Where brachiocephalic access is required, it can be achieved percutaneously via the right brachial or axillary route or open exposure of the right common carotid artery. Access to the left carotid artery is obtained by surgical exposure. Anatomically, increased arch angulation, minimum length for seal and irregular landing zone demand good stent design and material for adequate conformability with minimal change in aortic stiffness. Excessive mural thrombus and calcification together with proximity to the aortic valve increase the risk of embolisation. Careful and minimal wire and device manipulation is essential. Air emboli from the delivery system of the stent graft are a risk factor for stroke, and CO^2^ flushing has been proposed to prevent that risk [6]. However, more evidence is needed to prove that combined CO^2^ and saline flushing helps preventing air emboli before routine application of this technique is recommended. The risk of early mortality is higher in Ishimura zone 0 landing as compared to zone 1 or 2 [7]. Please see Fig. 1 for details about the Ishimura zones in the aortic arch [8]. It is important to perform the procedure using advanced imaging to avoid excessive radiation, reduce contrast volume and ensure accurate device placement and vessel cannulation. Transoesophageal echocardiogram can be an adjunctive tool during endovascular cases for guidewire placement in the dissected aorta or endoleak assessment [9]. Various monitoring tools have been used in open surgery for assessment of cerebral or spinal cord ischaemia including near infra-red spectroscopy, electroencephalography and motor or sensory evoked potentials. Evidence is lacking for their use in endovascular arch repair, but they can be used in selective endovascular cases (depending on the resources, the urgency of operation and the extent of the coverage of the aorta) [10]. Please see the fact sheet page for important anatomical parameters for endoluminal case planning.

## Endovascular Treatment Modalities

### Hybrid Repair

**Fig. 2:**
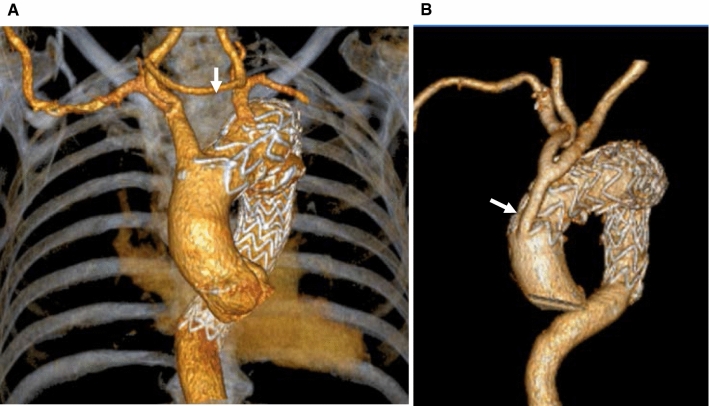
Hybrid arch repair. (**A**) Volume rendering frontal view of the aortic arch showing carotid-carotid cross over (arrow). (**B**) Neoinnominate surgical graft with bifurcation to the native innominate and left common carotid (arrowhead)

Hybrid repair of aortic arch pathology involves replacing one, two or all three supra-aortic vessels (debranching of the supra-aortic vessels) to create a sealing zone of healthy aorta of at least 2 cm in length, followed by thoracic endovascular aortic repair (TEVAR) stent placement in the arch, either staged or simultaneously. Several options exist for performing debranching including carotid to carotid bypass, left carotid to left subclavian artery (SCA) bypass or total arch debranching with fashioning of neoinnominate artery (see Fig. 2). Total arch debranching requires median sternotomy, and the graft is taken from the proximal ascending aorta. A staged procedure is our preferred option with debranching done first, followed by the TEVAR stent a week or two later. Arch disease involving zone 2 or 3 is better suited for hybrid repair with extra-anatomically carotid to carotid or left carotid to left SCA bypass, respectively. For arch diseases involving zone 0 or 1, total debranching of the arch vessels followed by endovascular stent landing in zone 0 is recommended. The hybrid procedure has the advantage over open arch repair in high-risk patients as there is no aortic cross-clamping involved and no requirement for cardiopulmonary bypass in most cases. Endografts available for this purpose are those used in descending aortic diseases, including Relay® PLUS (Terumo Aortic US Bolton Medical Inc), Conformable GORE TAG® Thoracic Endoprosthesis (W. L. Gore & Associates, Flagstaff, AZ, USA), Zenith Alpha Thoracic endovascular stent (COOK medical, Bloomington, IN, USA), and Valiant device (Medtronic, Minneapolis, MN, USA). The stroke risk following hybrid repair is significant reported at 28.6% in one study [11]. A meta-analysis involving 1,186 patients who underwent aortic arch hybrid repair reported mortality, stroke and spinal cord ischaemia rates of 10.8%, 6.9% and 6.8%, respectively [12]. The risk of spinal cord ischaemia is higher in cases of extended thoracic coverage. Maintaining a good peri-operative blood pressure, performing hybrid procedures in stages and revascularisation of the left SCA are considered to reduce the risk of spinal cord ischaemia. Dissection of the supra-aortic vessels carries risks of nerve injury (phrenic and vagus nerves) and lymph leaks. The presence of heavy calcified plaque, mural thrombus in the arch, and inadequate length of landing zone are some of the limitations for hybrid thoracic aortic stenting.

### Total Endovascular Approach

Technologies have evolved over the last decade and, in selected cases, can now provide a complete endovascular solution for aortic arch diseases in patients who are at high risk for open surgery. The surgery duration and the length of stay in the intensive care unit are significantly less in endovascular group as compared to open surgical repair [13]. Total endoluminal strategies involves custom-made devices with fenestration, scallop, inner branched endografts and parallel (chimney) endografts.

### Fenestrated/Scallop Endografts

**Fig. 3:**
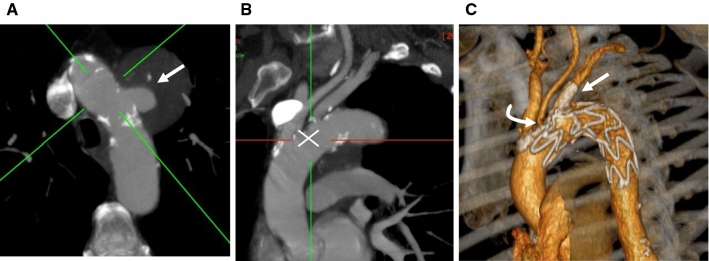
Stented fenestration and scallop. (**A**) Axial maximum intensity projection showing saccular aneurysmal penetrating aortic ulcer (arrow) in zone 2. (**B**) Oblique sagittal view with the white cross at the proximal edge of the ulcer. (**C**) Oblique volume rendering image post-stented fenestration (white arrow) and scallop to the left common carotid artery (curved arrow)

**Fig. 4:**
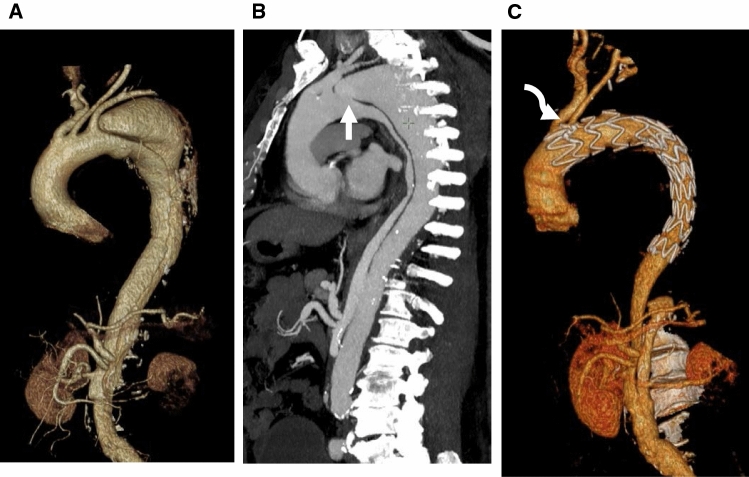
A patient with chronic type B aortic dissection treated with scallop stent graft (**A**) volume rendering oblique image showing aneurysmal dilatation, mainly at the distal arch level. (**B**) Maximum intensity projection showing the extent of the dissection which involves the origin of left subclavian artery (arrow). (**C**) Post-scallop stent graft insertion (curved arrow) with complete thrombosis of the false lumen down to the diaphragm level

**Fig. 5:**
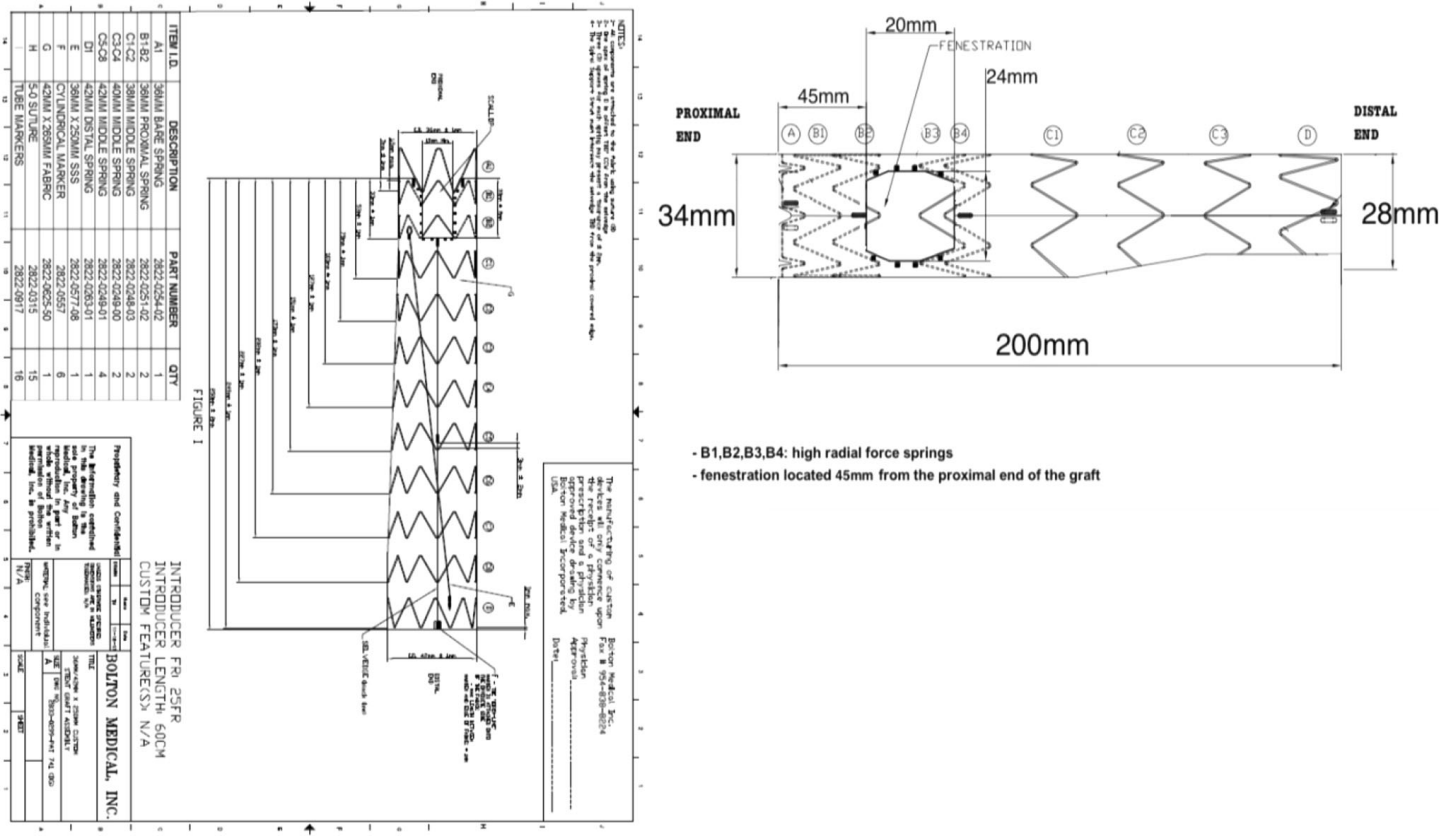
A schematic illustration of a scalloped/ fenestrated Relay ® (Terumo Aortic Bolton medical Inc.) endograft

Custom-made fenestrated or scallop stent grafts are manufactured using the standard stent graft platform. The fenestration or scallop is placed against the target vessels, extending the proximal landing zone without compromising the great vessels. They are suitable in cases where disease involves zone 2 and 3. Fenestrated arch devices have either single fenestration or a combination of fenestration and scallop, or a scallop alone (see Figs. 3 and 4). Figure 5 shows the schematic illustration of the scalloped and fenestrated endograft. The aorta must be non-aneurysmal at the level of fenestration or scallop and the ostia of the vessels at the outer curve of the arch should be along one line. The pre-curved nitinol cannula of the delivery system allows spontaneous orientation of the device. Commercially available devices are the Zenith fenestrated arch graft (COOK Medical, Bloomington, IN, USA) and Relay ®(Terumo Aortic US Bolton Medical Inc.). The Cook Fenestrated endograft has been reported safe and technically feasible with mortality up to 9%, stroke risk of 9%, temporary spinal cord ischaemia rates of 7% and early reintervention rates of 7% [14]. Please see Table 1 for the detailed results of some of the studies on endoluminal techniques. The Bolton Relay® scallop and/or fenestrated endograft can be used in any zone and has shown high technical success and low 30-day mortality. In two small single-centre series, the incidence of stroke ranged from 0–14% and endoleak from 10–19%. There was no evidence of migration or branch occlusion [15, 16]. The Dutch registry of 23 proximal scallop patients reported a 91% technical success rate. The incidence of clinical stroke and paraplegia was 4%. There was 2/23 and 1/23 type 1 and type 2 endoleaks, respectively, with no aneurysm-related death or growth over a mean follow-up time of 9 months [17]. A large unpublished series of 40 patients from our centre with a median follow-up of 2 years (range 1–8 years) reported on 14 cases with scallops and fenestrations in zones 0 and 1, and 26 patients in zone 3 and 4. No migration or vessel occlusion occurred over the period of follow-up with an 8% late reintervention rate to treat two cases of type 1a and one case of type 2 endoleaks. The authors consider fenestrated and /or scallop grafts in cases with saccular aneurysm along the inner curve of the arch, or cases with borderline length landing zone where proximal extension and inclusion of one or sometimes two great vessels will obviate the need for hybrid repair. The deployment of fenestrated or scallop endografts is relatively simple due to the pre-curved inner cannula, allowing self-orientation of the device with little manoeuvring by the operator.

**Table 1:** Highlighting the main results of some of the major studies on endoluminal techniques or aortic arch pathology

Authors, year, journal	No of patients/type of study	Device used	Main findings	Follow-up
Haulon et al. 2014, J Thoracic Cardiovasc. Surgery [21]	38 Multicentre Retrospective cohort Indications- Aortic arch dilatation > 5.5 cm unfit for surgery	Custom made double inner side branch aortic arch endograft – COOK medical (Bloomington, Ind) Branches to brachiocephalic and left carotid	Technical success—84.2% 30-day Mortality—13.2% Total endoleaks—28.8% Type 1–13.2% Early secondary intervention—10.5% Stroke—15.8% (minor stroke—10.5%) Spinal cord ischemia – 2.6%	Median FU-12 months Late re intervention- 9.1%
Verscheure et al. 2019, Annals of Surgery [34]	70 Multicentre Retrospective cohort Indications- Previously repaired type A dissection. Post-dissection arch aneurysm > 55 mm	Custom made Double inner side branch aortic arch endograft – COOK medical (Bloomington, Ind) – three inner branches	Technical skills—94.3% In hospital mortality and stroke—4% Early re intervention—17.1%, 2/12 for Endoleak (one type 2 and one type Ic)	Median FU- 301 Late re intervention -28.6%, 9/20 (12.8%) for Endoleak (7 for Type 1A) Late mortality 11.4%
Spear et al 2016, Eur Journal of Vas and Endovascular Surgery [35]	27 Multicentre Retrospective cohort Indications- Aortic arch dilatation > 5.5 cm unfit for surgery	Custom made Double inner side branch aortic arch endograft—COOK medical (Bloomington, Ind) two inner branches	Technical success—100% No death Major stroke—7.4% Minor stroke—3.7% Transient spinal cord ischemia—7.4% Re intervention—14.8%	Median FU 12 months Late re intervention 7.4% Endoleaks 11.1% (all type 2)
Yokoi et al. 2013, Journal Thoracic Cardiovas. Surgery [36]	383 multicentre Retrospective cohort Indication- Degenerative arch aneurysm, aortic dissection and others	Precurved customised for each patient with different types of stent and graft fenestrations Zone 0 in 363 patients	Technical success—99.2% 30-day mortality -1.6% Initial success (no type 1 or 3 endoleaks)—95.8% Stroke—1.8% Permanent paralysis—0.8% Retrograde dissection—0.8%	
Tsilimparis et al. 2020, JVS [14]	44 patients, Retrospective cohort Indication-post-dissection aneurysm, degenerative aneurysm, PAU	Custom made fenestrated endograft COOK medical (Bloomington, Ind) Bridging stents used Zone 0 sealing—27% Zone 1 sealing—62%	30-day mortality 9% Major stroke 7% Minor stroke 2% Temporary spinal cord ischemia- 7% Early re intervention—7% Retrograde dissection -1	Overall survival at 2 years 72% Late endoleaks: Type 1B—6 Type 3—1 Type 2—2 10 more late re intervention
Ali Alsafi et al. 2014, JVS [15]	21 patients, Prospective cohort Indications- Thoracic aneurysm involving zone 3/4	Custom made Bolton Relay Scalloped stent graft (Relay NBS; Bolton Medical Barcelona Spain) Hybrid repair—8 Scalloped only—13	100% technical success (no type 1 endoleak) 30—day mortality—5% Stroke—3/21 Paraplegia—1 (No death in Scalloped without extraanatomical group One stroke in scalloped only group)	Median FU 36 weeks Type 2 endoleaks in 3 patients at 52 weeks—one required re intervention One type 3 endoeleak
Bosiers et al. 2016, Ann Thoracic Surgery [27]	European multicentre registry for Chimney/ Snorkel 95 patients Indications Degenerative aneurysm, post-dissection aneurysm, PAU, previous endoleak type 1a	Multiple aortic devices; Gore—60% COOK Zenith—20% Chimney Stents; Balloon expandable stents—28.4% Self-expandable stents—59.8% Bare metal stent—11.8% Left SCA chimney – 61.8% Left CCA chimney—23.5% Brachiocephalic trunk chimney—12.7%	Emergency repair—48.4% Technical success—89.5% 30-day mortality 9.5% Type 1a Endoleak 10.5% (50% resolved in 30 days) Type 1B Endoleak—4.2% Type 2 Endoelak—16.8% Major stroke—2% (Overall—4.2%) Re intervention—5.2%	Freedom from intervention—96.5% at 1 year and 88.6% at 5 years Gore with self-expanding and Valiant with balloon expandable stents effective and associated with low risk of gutter leak

### In Situ Fenestration of the Standard Stent Graft

This is an off-label technique used in emergency settings, in which a fenestration is made in the stent graft by mechanical means or laser technique. The fenestration is sequentially dilated with balloons and finally, a stent is placed across the fenestration into the target vessel, usually from peripheral access in a retrograde manner. Qin et al. reported laser-guided fenestrations in 24 cases of aortic arch stent grafts with 16/24 fenestrations for left SCA alone and only 2 for all three arch vessels. Technical success was reported at 95.8%, with no endoleak or stroke [18]. The main risks associated with this technique are technical failure which can damage the graft and cause type 3 endoleak and risk of end-organ ischaemia (particularly brain ischaemia) due to the time taken in performing the fenestration. No long-term data are available and the risk of damaging the stent graft has not been extensively studied [19].

### Arch Branched Grafts

**Fig. 6:**
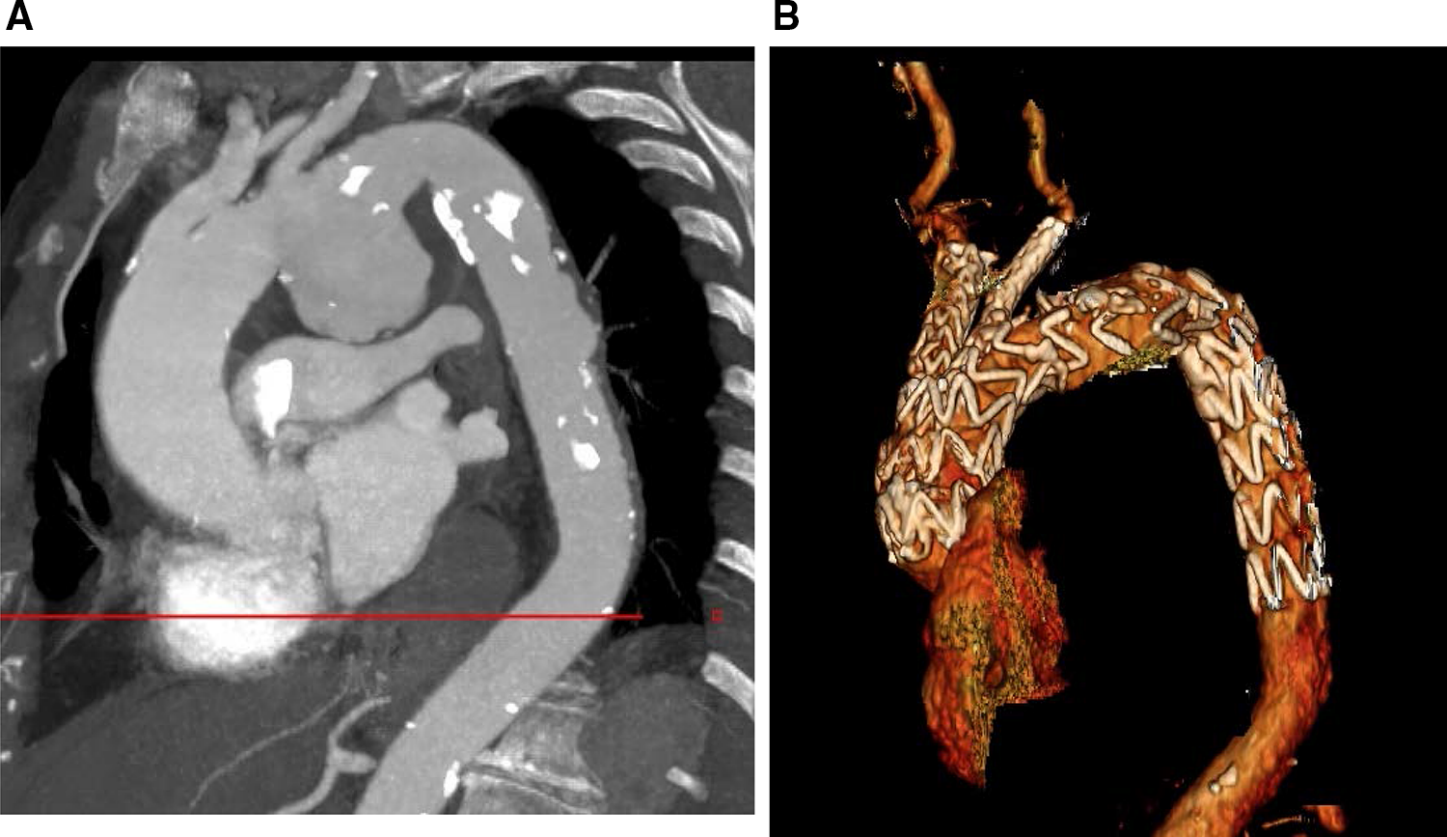
Branched arch stent graft. (**A**) Oblique sagittal maximum intensity projection of the aortic arch showing 6.5 cm saccular aneurysm involving the inner curvature of the aorta. (**B**) Volume rendering image post-double branch stent graft insertion

**Fig. 7:**
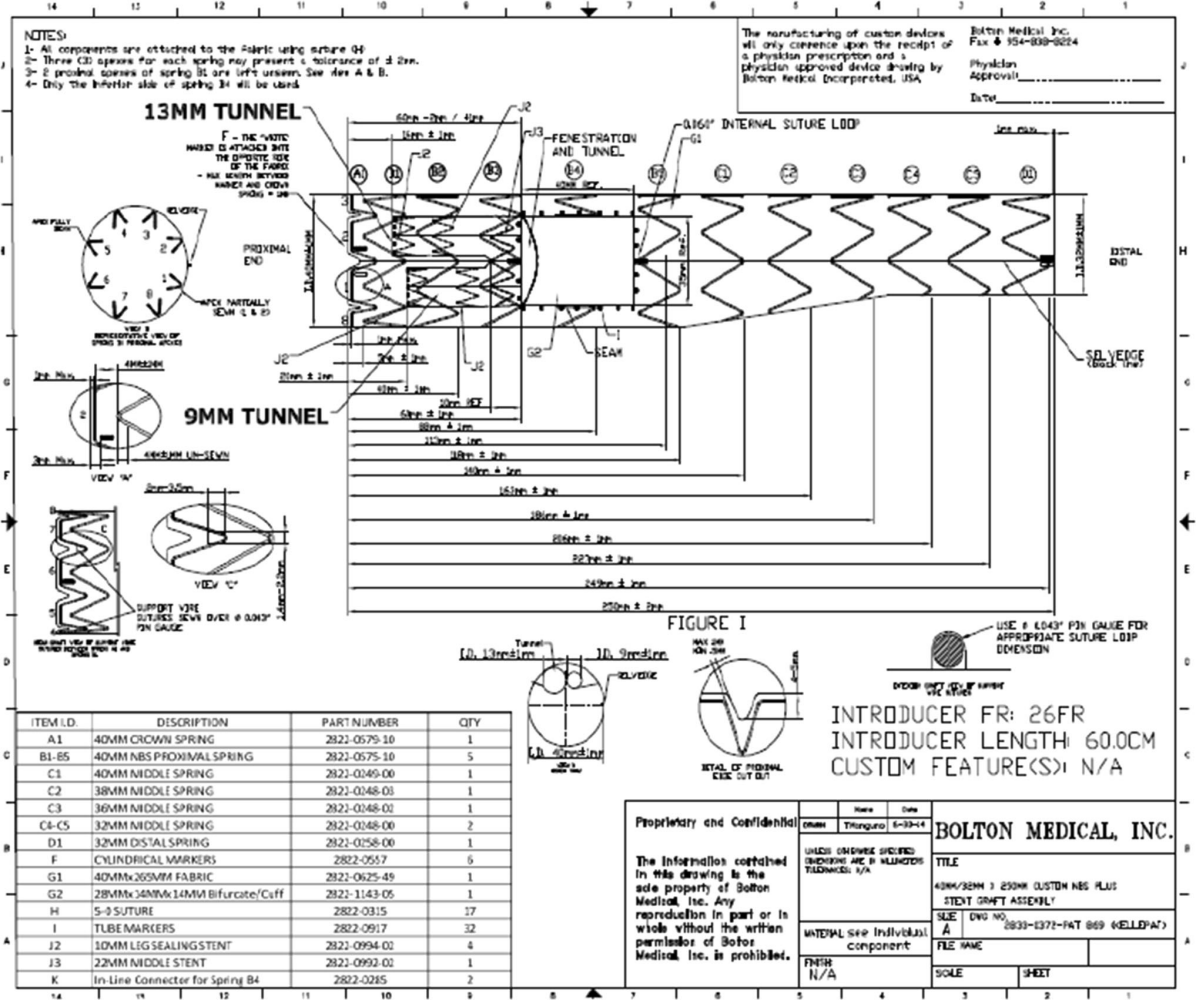
A schematic illustration of a Relay ® (Terumo Aortic Bolton medical Inc.) branched endograft with inner branches

Two main designs of branched endografts are available so far in the market: one device with two antegrade internal tunnels, and a second design with a retrograde single branch. The first type from Cook Medical (Cook Medical, Bloomington, IN, USA) and Bolton Relay Plus (Terumo Aortic, Sunrise, FL, USA) has tunnels with antegrade flow towards the brachiocephalic trunk and left carotid. Access for the left carotid branch is gained retrogradely from left carotid puncture, while access to the innominate branch is obtained retrogradely from either the right common carotid or axillary artery. Depending on the stent manufacturer and native vessel diameter, the profile of the bridging stent for the brachiocephalic inner branch ranges from 9 to 14 F and cutdown is usually needed. The profile of the bridging stent for the left carotid inner branch is usually 6-8F, and open access is usually obtained over the left common carotid artery. The main thoracic stent graft is deployed from the femoral artery access over a stiff wire, the tip of which is placed in the apex of the left ventricle. A pigtail angiographic catheter is placed near the sinotubular junction. Rapid pacing or an inferior vena cava balloon is required to reduce the cardiac output down during deployment. The profile of different branched stent grafts is provided in Table 2. The delivery systems of both the COOK and Bolton Relay devices are similar and come with a pre-curved nitinol inner cannula which helps orientate the branches along the outer curvature of the aortic arch. The Cook graft delivery system also has multiple trigger wires which helps in controlled deployment. The branched graft usually lands further proximally in the ascending aorta than the fenestrated graft (see Fig. 6). Please see Fig. 7 for a schematic illustration of arch branched endograft with two antegrade tunnels. Arch branch endograft is suitable for aortic pathology where involving zones 0, 1 and sometimes 2. The limitations of these devices are a required landing zone length of at least 4.5 cm in the ascending aorta, a maximum native diameter vessel of 38 mm, and the inability to use them in the presence of metallic aortic valve. Please see Previous surgical repair for type A dissection can provides an excellent landing zone for branched endograft, as this eliminates risk of retrograde type A dissection completely provided that the interposition graft has an adequate length. A series of 30 consecutive patients with a range of arch pathologies treated with branched stent graft from Cook reported 90% technical success, 7% neurological events, 30-day mortality of 10% and reintervention of 20% [20]. These results showed improved outcomes when compared with a previous multicentre study of 38 patients that reported 13% mortality and a stroke rates of 16% (Please see Table 1) [21]. Recently, a single centre experience of custom-made inner branched arch endograft with two internal branches (Cook Medical, Bloomington, IN, USA) and left-sided carotid to SCA bypass of 54 consecutive patients has reported a technical success rate of 98% and a 30-day mortality and major stroke incidence of 5.5% and 5.5%, respectively [22]. No retrograde type A dissections or cardiac injuries were observed. Limited experience was reported for three-vessel custom-made endoprosthesis. Similar promising results have been reported with the Bolton Relay Plus branched arch endograft (Terumo Aortic, Sunrise, FL, USA). Czerny et al. reported 15 consecutive patients treated with double-branched stent graft achieved 100% technical success. However, neurological events, a mixed of disabling and non-disabling stokes, were observed in around 20% of patients [23]. In-hospital mortality was 7%, with 100% aorta-related survival over median follow-up of 263 days.

**Table 2:** Table explaining the details of the various branched endovascular stent grafts used in aortic arch

Device manufacturers	Stent material/graft material	No. of branches	Aortic graft diameter in mm	Branch graft diameter in mm	Main device profile (F)
PTMC Institute (Kyoto, Japan)	Nickel Titanium/ Dacron	1–3	18–46	8–20	20–24
Bolton Medical (Sunrise Flo USA)	Nitinol/ Polyester	1or 2/antegrade inner branched	46	20	26, 25
Cook (Bloomington, IN, USA)	Nitinol/ Polyester	2 or 3 2 antegrade and 1 retrograde inner branched	38–46		22–24
S&G Biotech Inc (Seongnam Korea)	Nitinol/ Polyester	2	44	18,10	21,18
Microport Medical Co Limited (Shanghai, China)	Nitinol/ Polyester	1	28–40 30–34	7.5–14 10–16	22 18–24
Medtronic Vascular (Santa Rosa, CA, US)	Nitinol/ Polyester	1	30–46	10–14	24–25
WL Gore (Flagstaff AZ, US)	Nitinol/PTFE	1 retrograde inner branched	21–53		
Endospan (Herzlia, Israel)	Nitinol/PTFE	1	36–43	14–20	20

*PTFE* Polytetrafluoroethylene

The Inoue device (PTMC Institute, Kyoto, Japan) is a custom-made all-in one stent graft with aortic stent section and branched section sewn together. It comes with a mixed number of branches. The largest series of 89 patients treated with the Inoue device showed high technical success and a low 30-day mortality of 4.5% but high stroke rate of 16% [24].

A different branched design is available from Gore & Associated (Gore Medical Flagstaff, AZ, USA), whereby a retrograde single branch is assembled into the Conformable TAG device. The Gore graft is designed for zone 0 or 2. Patel et al. reported early results of a multicentre study of 24 patients with aneurysmal disease treated with a single branch stent graft to the left SCA. The technical success and survival rates were 100% with no reported stroke in this small and highly selected group [25]. Another single retrograde side branch endograft, Valiant Mona LSA (Medtronic Inc Santa Rosa CA USA), has also been investigated. These are usually suitable for zone 2 or more proximal zones where they are combined with extra-anatomical bypass. Both the Gore and Valiant systems have a preloaded catheter inside the branch, which is threaded and requires snaring from the brachial, axillary or carotid access in the arch and deployment over the through-and-through wire. Please see Table 2 for the details of different arch branched devices.

Overall technical results are particularly good with both branched and fenestrated arch devices. These should be considered in patients who are not fit for open repair. The arch branch technique provides a durable repair as the landing zone is in the ascending aorta. However, the procedure itself is complex and not possible if the ascending aorta is aneurysmal. In our experience, we consider arch branch technology for pathology in zones 0–2, when the great vessels are not suitable for scallop or fenestration because of vessel alignment or inadequate landing zone, and unfit patients with reasonable life expectancy.

### Chimney Grafts/Parallel graft/Periscope

**Fig. 8:**
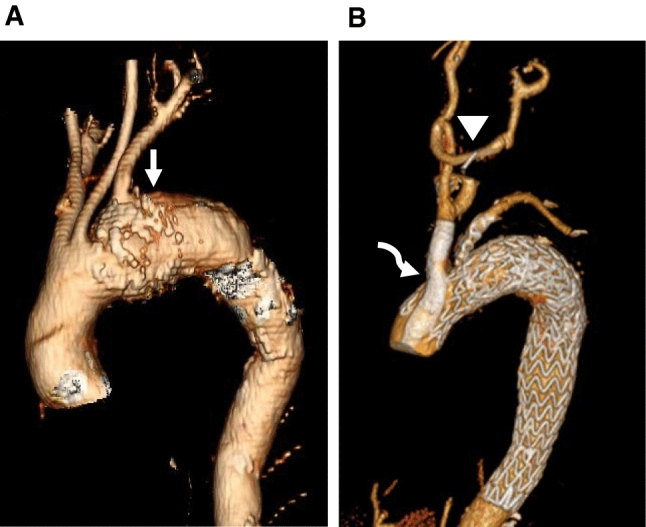
Single chimney stent. (**A**) An elderly patient presented with symptomatic 6 cm saccular aneurysm in zone 2 ((white arrow). (**B**) Post-right carotid to left carotid bypass (arrowhead) and single chimney stent in the innominate artery (curved arrow) and TEVAR. The left subclavian artery was embolised with closure device

The concept of parallel chimney stents was initially described as a “bailout” technique after inadvertent renal artery coverage during endovascular abdominal aortic repair [26]. The direction of flow is antegrade in a chimney graft and retrograde in periscope technique. The main aortic stent is inserted via femoral access into the pre-defined place in the thoracic aortic arch. Via retrograde approach from the common carotid or arm access (axillary or brachial), the chimney grafts are positioned parallel to the aortic stent-graft and protruded around 1–2 cm proximal to the main aortic stent with the distal stent segment sitting in the branched vessel. The main TEVAR and the chimney stent are deployed sequentially and may be moulded with a balloon (see Fig. 8). For carotids chimney stent, access is achieved via surgical cutdown in the neck. Commonly used stents for the chimney are Fluency (C.R. Bard, Inc, Murray Hill, NJ, USA), Viabahn® (W.L. Gore, Inc., Flagstaff, AZ, USA), Atrium (Maquet Getinge Group, Inc., Germany) and Bentley. For a large diameter innominate artery, iliac endograft limbs (such as Zenith Flex® TFLE/ZSLE; Cook Medical, Inc., Bloomington, IN, USA) can be used as chimney stents. It has been technically feasible to use this technique into supra-aortic branches parallel to the main aortic stent graft for aortic arch diseases [27, 28]. Balloon expandable stents are preferable to self-expanding due to their accurate deployment and higher radial force [29]. Chimneys are mostly performed for acute emergency reasons such as dissection, IMH and pseudoaneurysm, with the commonly used proximal landing zone site is in zone 2, and frequently done for a single vessel [30]. This technique is relatively cheaper to use, and it is perceived as less technically demanding [7]. Other examples of parallel graft are periscope and sandwich techniques. A periscope graft is inserted from femoral access into the supra -aortic vessel, usually the left SCA, or inserted retrogradely from the SCA vessel into the descending aorta. The periscope endograft sits parallel to the main aortic graft and extends distal to the thoracic aortic stent graft. The sandwich technique involves placing the target artery stent between two overlapping thoracic aortic stents.

O’Callaghan retrospectively compared custom made fenestrated endograft (*n* = 15) against non-custom-made chimney stent (*n* = 18). In hospital mortality was higher in non-custom group (7 vs 18%). There was a trend noted favouring better durability of fenestrated graft for sealing and re intervention rates but results were not statistically significant [31]. One of the most debated down sides of the chimney stent graft is the risk of type 1 endoleak and endotension due to the gutter effect between the aortic endograft and the chimney graft, raising doubts over its durability. It has been suggested that the risk is higher when the pathology involves the outer curvature of the aorta than the inner curvature of the aorta [32]. A European multicentre registry on the chimney or snorkel technique reported the outcomes of 95 patients. Technical success was achieved in 89.5% of cases, with 9.5% 30-day mortality and 10.5% type 1a endoleak. Freedom from intervention at 5 years was 88.6% [27] (please see Table 1). A meta-analysis of 11 publications involving 373 patients who underwent chimney endografts for their aortic arch disease (reported overall technical success rates of 91.3%, overall estimated proportion of early type 1a endoleak of 9.4% and retrograde type A dissection of 1.8%. The reported pooled 30-day mortality was 7.9%, with a reintervention rate of 10.6% and major strokes rate of 2.6% [30]. No long-term data exists regarding the durability of the chimney graft in the aortic arch. An overlap of at least 2 cm between the chimney and the thoracic stent-graft, adequate oversizing of the thoracic stent-graft (30%) except in acute dissection and kissing balloon angioplasty in the chimney and aortic stent-grafts simultaneously, with rapid inflation/deflation and moderate inflation pressure may decrease the risk of endoleak. In our experience, we reserve the use of chimney for emergency cases and as a bailout in inadvertent cover of a great vessel. We tend to oversize the TEVAR stent by at least 15–20%, attempt to extend the tunnel length more than 25 mm and limit the number of chimneys to one whenever possible. The double chimney technique carries much higher rates of complications including type 1 endoleaks and its use is controversial [33].

Please see the fact sheet for some of the important papers underlining the key evidence regarding the endoluminal techniques in aortic arch repair.

## Complications Associated with Endoluminal Techniques

Generic complications associated with endoluminal technique for the aortic arch include the risk of arrhythmias and myocardial ischaemia due to the close proximity of the wire or sheath to the coronary ostia and left ventricles. Cardiac complications including acute coronary syndrome and arrhythmias have been reported in the range from 4.3 to 7.9% [21, 34]. Cardiac perforation by the stiff wire is a recognised complication and must be avoided by careful wire manipulation. Access site complications are not uncommon and occasionally requires re intervention for pseudoaneurysm and arterial repair. Verscheure et al. reported 11.4% access site complications requiring intervention [34]. Temporary renal impairment is common, and care should be taken in the use of contrast. Moreover, any nephrogenic medications such as metformin should be withheld for 48 h after the procedure. Wound infection, nerve injury and lymph leak are rare but known complications associated with surgical cutdown.

## Conclusion

Endoluminal techniques should be considered for patients with technically feasible aortic arch disease who are not suitable for open repair. A collaborative multidisciplinary team-based approach must be taken in choosing individualised treatment for the patient based on patient’s pathophysiology, anatomical features of the arch and the experience of the team. Custom-made branched, fenestrated or scalloped endoluminal techniques should be preferred over the chimney technique for elective repair of the aortic arch. The chimney technique should be reserved as a bailout approach for emergency cases. We may see developments in new technologies, including in situ fenestration techniques, over the coming years.
